# Tick Infestation of Eyelid: Two Case Reports

**DOI:** 10.4274/tjo.43660

**Published:** 2016-10-17

**Authors:** Aslıhan Uzun, Mustafa Gök, Murat Doğan İşcanlı

**Affiliations:** 1 Ordu University Training and Research Hospital, Department of Ophthalmology, Ordu, Turkey; 2 Ordu University Training and Research Hospital, Department of Emergency Medicine, Ordu, Turkey

**Keywords:** Eyelid, tick, Crimean Congo hemorrhagic fever

## Abstract

Tick infestation of the eyelid is a rare but serious condition that can lead to Crimean-Congo hemorrhagic fever. In this report, we describe two cases who presented with tick infestation of the eyelid. Neither patient developed systemic disease or adverse sequelae after tick extraction. Complete mechanical removal of ticks located on the eyelid with blunt forceps is a safe and effective treatment method.

## INTRODUCTION

Ticks are ectoparasites which live by hematophagy of mammals, birds and reptiles, and consequently act as vectors of various diseases. Tick infestation has gained attention in recent years due to Crimean-Congo hemorrhagic fever (CCHF), a potentially fatal tickborne disease. Ticks have also been associated with localized lesions resembling erythema chronicum migrans, foreign body granuloma, lymphoid hyperplasia and tick-related alopecia.^1^

Tick infestation of ocular tissues is a rare occurrence. In this report, we describe two cases that were treated in our clinic just one week apart, one who presented complaining of a tick bite on the eyelid and another in which a tick was found on the eyelid during routine examination.

## CASE REPORTS

### Case 1

**Figure 1:**
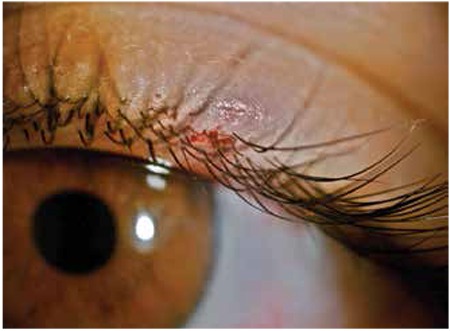
Tick infestation of the left superior eyelid of a 15-year-old female patient. Mild hemorrhage and ecchymosis of the eyelid are apparent. The tick’s body was severed due to inappropriate extraction method

Our clinic was consulted by emergency services regarding a 15-year-old female patient who presented complaining of a tick bite on her left upper eyelid. The patient’s family reported that they had been in a hazelnut orchard the previous day and the patient had slept under a tree. That evening she had complained of itching on her left upper eyelid. Her family noticed a tick on her eyelid and took her to emergency services. The patient was referred to our clinic following unsuccessful attempts to remove the tick from the patient’s eyelid in the emergency services department. On slit-lamp examination the tick was visible on the left superior eyelid just anterior to the eyelash line (Figure 1). We also noted edema and hyperemia of the eyelid, and mild hemorrhage and ecchymosis resulting from tissue damage incurred during attempts to remove the tick. The body of the tick had also been severed during these attempts; we carefully removed the remaining tick parts using toothless forceps with curved, blunt medium tips. Complete removal of the tick was confirmed by slit-lamp examination and 10% povidone iodine was applied to the area. At time of presentation, the patient’s white blood cell count was at the upper limit of the reference range (10.7x10^3^/µl) and eosinophil level was elevated (1.03x10^3^/µl). The patient was prescribed topical tobramycin/loteprednol etabonate eye drops (instilled four times a day) and fusidic acid eye drops (twice a day). Hemogram, biochemical and serologic tests were ordered to determine the presence of CCHF, Lyme disease, tularemia or Q fever. The patient’s whole blood, prothrombin time (PT), partial thromboplastin time (PTT), international normalized ratio (INR) and other biochemical parameters were within reference ranges. The following day, her eyelid edema and hyperemia had regressed and no additional pathologic findings had developed. The patient was called for follow-up one week later due to the risk of CCHF, but no pathology was detected in her PT, PTT, INR tests or the results of the other serologic tests ordered for CCHF. After one week the patient’s edema and hyperemia had resolved and the site of the tick bite had healed with no scarring.

### Case 2

**Figure 2:**
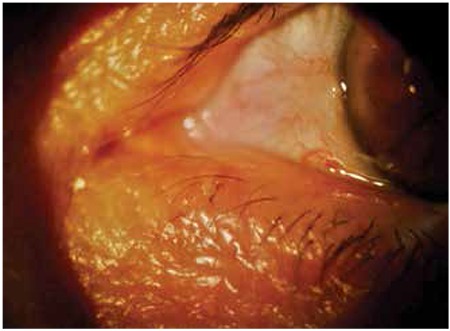
Tick infestation of the left inferior eyelid of a 66-year-old female patient

A 66-year-old female patient presented to our clinic with complaints of itching and stinging in her right eye. During routine examination a tick infestation was discovered incidentally on the patient’s left lower eyelid (Figure 2). However, no accompanying findings were apparent in the eyelid or eye. It was learned that the patient lived in a rural area, but she reported no contact with any animals. The tick was carefully removed using toothless forceps with curved, blunt medium tips. Complete removal of the tick was confirmed by slit-lamp examination, and 10% povidone iodine was applied to the area. The patient was prescribed topical tobramycin/loteprednol etabonate eye drops (instilled four times a day) and fusidic acid eye drops (twice a day). Hemogram, biochemical and serologic tests were ordered to determine the presence of CCHF, Lyme’s disease, tularemia or Q fever. The patient’s whole blood, PT, PTT, and other biochemical parameters were within reference ranges. The patient was invited for follow-up one week later due to the risk of CCHF; the site of the tick bite had healed without scarring or sequelae. No pathology was detected in the PT, PTT, INR tests or the results of the other serologic tests ordered for CCHF.

## DISCUSSION

Ticks are ectoparasitic vectors of serious, potentially life-threatening diseases such as CCHF, Lyme disease, tularemia, and Q fever.^2^ The subtropical climate of Turkey provides a suitable environment for a wide variety of ticks. In addition to the climate, the breeding of livestock and uncontrolled application of pesticides create ideal conditions for ticks.^3^ Ticks may mimic a mass on the eyelid, especially if located at the meibomian gland orifices. Previous reports of ocular tick infestation have documented cases with ticks located on the eyelid or conjunctiva.^3,4^

Removing ticks whole from the affected tissue is of utmost importance in the prevention of tickborne diseases and possible abscess, granuloma, or other local lesions. Therefore, ticks should be removed immediately and carefully from affected tissue. Human and animal studies have demonstrated that the risk of disease transmission and infection increases after the first 24 hours of tick infestation and is especially high after 48 hours.^5,6^ After removal of the tick and all its body parts, the patient should undergo all necessary blood and serologic tests and be monitored closely for local and systemic complications.

Various chemical and mechanical techniques have been recommended for the removal of ticks, and even surgical excision of the tick with surrounding tissue has been proposed. Ether, lindane shampoo, deodorized kerosene, and iodine solutions have been used to prevent the disintegration of the tick and facilitate whole removal. However, experimental studies have shown that it is not possible to remove ticks whole using chemical substances and also that the use of chemicals stimulates ticks’ salivation and increases the risk of disease transmission.^7^ Furthermore, the chemicals used irritate the eyes.

The careful, mechanical extraction of ticks using blunt, curved, medium point forceps is recommended as safe and effective.^6^ There is controversy concerning the administration of systemic antibiotic prophylaxis administration after tick bites. The Infectious Diseases Society of America does not recommend systemic antibiotic prophylaxis following a tick bite. According to the literature, however, local and topical antibiotics are used.

Both of our cases were exposed to ticks in rural areas. Using forceps with curved, blunt medium tips, we were able to safely remove the tick parts remaining after a failed removal attempt in emergency services in the first case and the intact tick in the second case. Blood and serologic tests necessary for diagnosis and follow-up of CCHF, Lyme’s disease, tularemia and Q fever were performed in both cases. We did not administer systemic antibiotics to our patients. Both the local tissue reaction of the 15-year-old girl and our 66-year-old female patient were successfully treated with combined topical steroid and antibiotic treatment after mechanical extraction of the tick. No signs of local or systemic disease were observed in either patient during follow-up.

In conclusion, ophthalmologists should be aware of possible local and systemic diseases that may arise after tick infestation of the eye and adjacent structures. Mechanical extraction of the whole tick using blunt forceps is a safe and effective treatment option. Patients should undergo blood and serologic tests for tickborne diseases and be monitored with clinical observation and follow-up.

### Ethics

Informed Consent: Informed consent was obtained from patients.

Peer-review: Externally and internally peer-reviewed.
